# 608. Evaluation of Safety of High-Dose Beta-Lactam Antibiotics in Patients with End-Stage Kidney Disease: A Retrospective Cohort Study

**DOI:** 10.1093/ofid/ofac492.660

**Published:** 2022-12-15

**Authors:** Jerald Varona, Alireza FakhriRavari, Abbas Hassan, Rosa Trieu

**Affiliations:** Loma Linda University School of Pharmacy, Loma Linda, California; Loma Linda University School of Pharmacy, Loma Linda, California; Loma Linda University School of Pharmacy, Loma Linda, California; Loma Linda University School of Pharmacy, Loma Linda, California

## Abstract

**Background:**

Beta-lactam toxicity is often underestimated in clinical practice given the insidious onset and complex clinical picture of patients. Renally impaired patients are at increased risk of toxicity due to drug accumulation, which may increase their morbidity and mortality. This study’s objective is to identify the rate of beta-lactam-induced toxicities in patients with end-stage kidney disease (ESKD) on higher than recommended doses of piperacillin-tazobactam, cefepime, or meropenem compared to patients on appropriate doses (Table 1).

**Figure:**
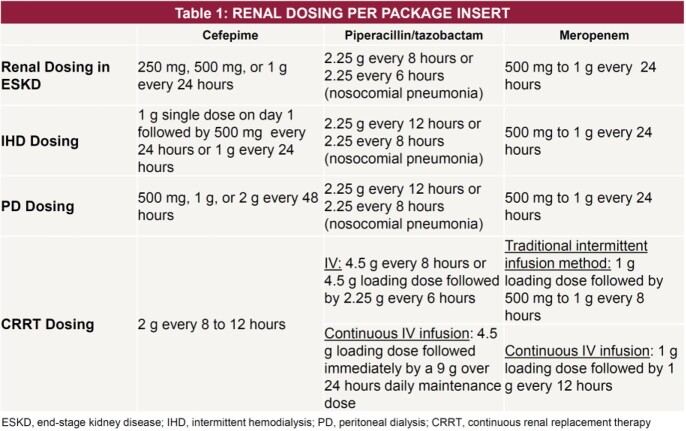

**Methods:**

This retrospective cohort study examined patients hospitalized at Loma Linda University Medical Center in Loma Linda, California, from January 2013 to June 2021. Patients 18 to 89 years old with ESKD via ICD-10 code, and who received piperacillin-tazobactam, cefepime, or meropenem for 48 hours or more were included. The primary composite outcome of beta-lactam toxicity consisted of neurotoxicity, hepatotoxicity, and hematologic toxicity. Secondary analyses of each toxicity were done. Statistical analysis was done using R software with a p-value < 0.05 considered significant. Multivariate logistic regression was done for variables with a p-value < 0.2 in bivariate analysis.

**Results:**

Eligibility criteria were met by 341 patients. Cohort 1 (N=193) received appropriate antibiotic doses and cohort 2 (N=148) received higher than recommended doses. Baseline characteristics were similar except for renal replacement therapy and meropenem use (Table 2). In cohort 1, 58.5% of patients experienced beta-lactam associated toxicity (Table 3), compared to 70.9% of patients in cohort 2 (Odds ratio [OR] 1.73, 95% confidence interval [CI] 1.07-2.81, p=0.02). The rate of neurotoxicity was 29.0% in cohort 1 compared to 42.6% in cohort 2 (OR 1.81, 95% CI 1.13-2.92, p=0.01). Receiving higher than recommended doses was an independent risk factor for developing any beta-lactam associated toxicity (OR 1.63, 95% CI 1.02-2.63, p=0.04, Table 4), including neurotoxicity (OR 1.63, 95% CI 1.02-2.62, p=0.04).

**Table 2:**
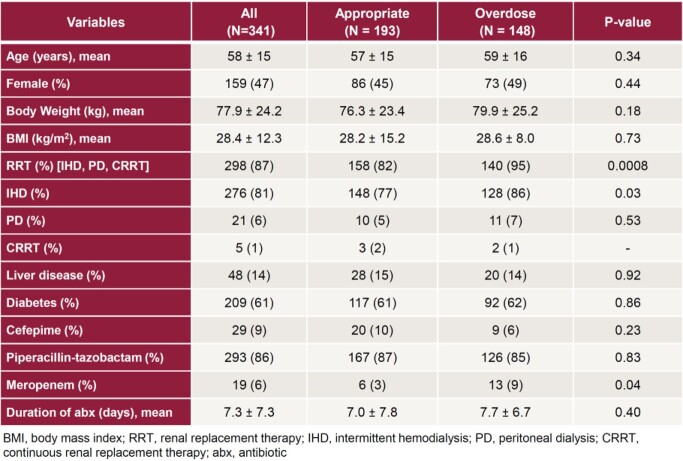
Baseline Characteristics

**Table 3:**
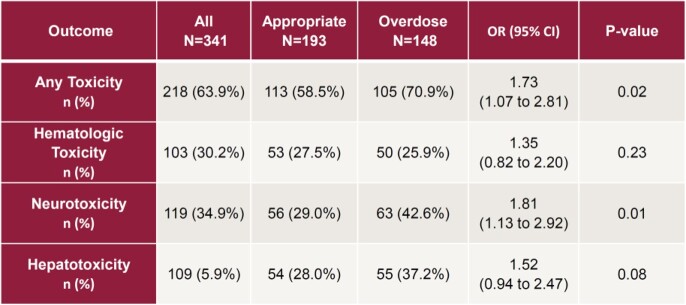
Primary and Secondary Outcomes

**Table 4:**
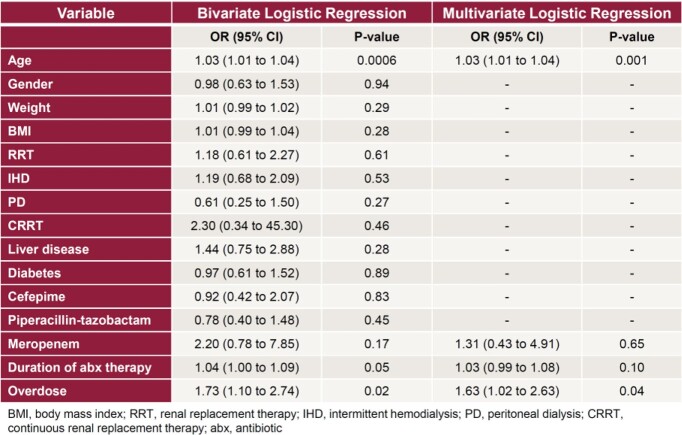
Logistic Regression Analysis for Composite Primary Outcome

**Conclusion:**

Patients with ESKD on higher than recommended doses per package insert of piperacillin-tazobactam, cefepime, or meropenem for at least 48 hours were significantly at higher risk of beta-lactam associated toxicity, specifically neurotoxicity.

**Disclosures:**

**All Authors**: No reported disclosures.

